# Associations of Dietary Inflammatory Potential, Dietary Diversity, and Micronutrient Adequacy with Rheumatoid Arthritis: A Case–Control Study from India

**DOI:** 10.3390/nu18193170

**Published:** 2026-09-26

**Authors:** Hemant Mahajan, Sailaja Pothana, Phani Kumar Devarasetti, Janhavi Pawar, Ranga Sreenivasa Reddy Kypa, Aditi Abhay Deshpande, Nikhita B. R. R. Boda, Venkat Raji Reddy Garlapatti, Srikanthbabu Perugu, Jag Jeevan Babu Geddam, Liza Rajasekhar, Devraj J. Parasannanavar

**Affiliations:** 1 Indian Council of Medical Research—National Institute of Nutrition, Hyderabad 500007, India; mahajan.h@icmr.gov.in (H.M.); shailaja_nin@yahoo.co.in (S.P.); rangalt.ranga@gmail.com (R.S.R.K.); mail2aad@gmail.com (A.A.D.); nikhitab.r@gmail.com (N.B.R.R.B.); gvenkatrajireddy1980@gmail.com (V.R.R.G.); geddambabuj@gmail.com (J.J.B.G.); 2 Department of Rheumatology, Nizam’s Institute of Medical Sciences, Hyderabad 500082, India; dphanimbbs@gmail.com (P.K.D.); lizarajasekhar@gmail.com (L.R.); 3 Specialized Dietetics, Sir Vithaldas Thackery College of Home Science, Mumbai 400049, India; pawarjanhavi2002@gmail.com; 4 Department of Kayachikitsa, Dr. B.R.K.R. Government Ayurvedic Medical College, S.R. Nagar, Erragadda, Hyderabad 500038, India; drperugu@gmail.com

**Keywords:** rheumatoid arthritis, diet, inflammation, nutrition assessment, nutritional status, anti-citrullinated protein antibodies

## Abstract

**Background:** Rheumatoid arthritis (RA) is a chronic autoimmune disease influenced by genetic and environmental factors, including diet. We examined the associations of dietary inflammatory potential, dietary diversity, and micronutrient adequacy with RA and RA-related autoantibodies in an urban Indian population. **Methods:** In this unmatched case–control study, 301 newly diagnosed RA cases and 314 healthy controls from Hyderabad, India, were enrolled. Dietary intake was assessed using a validated 131-item food frequency questionnaire (FFQ). Dietary inflammatory potential was estimated using the Dietary Inflammatory Index (DII), while dietary quality was assessed using the dietary diversity score (DDS) and mean probability of micronutrient adequacy (MPA). Rheumatoid factor (RF) and anti-cyclic citrullinated peptide (anti-CCP) antibodies were measured by ELISA. Multivariable regression models adjusted for age, sex, and BMI were used to assess associations. Population attributable fractions (PAFs) were estimated under a theoretical causal framework. **Results:** Compared with controls, RA cases had lower micronutrient adequacy, lower dietary diversity, and higher dietary inflammatory potential. Each one-unit increase in DII was associated with higher odds of RA (AOR 1.17; 95% CI: 1.10, 1.24), whereas each one-unit increase in DDS (AOR 0.73; 95% CI: 0.66, 0.81) and each 10-percentage-point increase in MPA (AOR 0.88; 95% CI: 0.83, 0.94) were associated with lower odds of RA. Associations with autoantibody concentrations were limited. Higher DDS, when analyzed as a continuous variable, was associated with lower anti-CCP concentrations, but not with RF. Continuous DII and MPA were not significantly associated with either anti-CCP or RF, and associations across categorical analyses were inconsistent. **Conclusions:** Poorer dietary diversity, lower micronutrient adequacy and a greater dietary inflammatory potential were associated with higher odds of RA. Associations with RA-related autoantibody concentrations were limited and inconsistent, and no significant associations with RF were observed. Given the case–control design, these findings should be interpreted as associations and require confirmation in prospective studies.

## 1. Introduction

Rheumatoid arthritis (RA) is a chronic systemic autoimmune disease characterized by persistent synovial inflammation, progressive joint destruction, disability, and increased mortality. It affects approximately 0.5–1% of the global population, occurs two to three times more frequently in women than men, and commonly manifests during middle adulthood [1,2,3]. The pathogenesis of RA involves genetic and environmental factors, with the HLA-DRB1 gene being strongly associated with disease susceptibility [4,5,6]. Environmental triggers, including cigarette smoking, infections, and dietary factors, interact with genetic susceptibility to influence disease onset and progression through immune dysregulation and inflammatory pathways [7,8,9,10]. Dietary intake plays a critical role in modulating inflammation, a central process in RA pathophysiology, through its effects on immune regulation, oxidative stress, gut microbiota, and inflammatory cytokine production [10,11,12].

Diets with anti-inflammatory properties, particularly the Mediterranean diet, have been associated with reduced disease activity, improved physical function, and symptom relief in patients with RA, whereas pro-inflammatory dietary patterns characterized by high intakes of processed foods, refined carbohydrates, saturated fats, and red meat (Western-style diets) may promote systemic inflammation and contribute to disease progression [10,11,13,14]. Rapid urbanization and the nutrition transition in India have resulted in increased consumption of energy-dense and animal-source foods, potentially contributing to chronic inflammatory conditions [15,16]. Western dietary patterns characterized by high intakes of saturated and trans fats, refined carbohydrates, processed foods, and an unfavorable omega-6 to omega-3 fatty acid ratio promote systemic inflammation and have been associated with an increased risk of rheumatoid arthritis [10,11,17,18]. While dietary influences on inflammation in Western populations are well-documented, there is a paucity of research on the role of traditional Indian dietary patterns in RA-related inflammation. Macronutrients and micronutrients modulate RA pathophysiology by influencing immune function, oxidative stress, cytokine production, and other inflammatory pathways [10,14,19].

Higher dietary fiber intake is inversely associated with inflammatory markers such as C-reactive protein (CRP), interleukin-6 (IL-6), and tumor necrosis factor-α (TNF-α), whereas Western dietary patterns characterized by low fiber and high consumption of processed and animal-derived foods are associated with greater systemic inflammation [10,20,21,22]. Micronutrients, including antioxidant vitamins (C, E, and B complex) and trace minerals such as magnesium, zinc, and selenium, modulate immune responses and oxidative stress, thereby exerting antioxidant and anti-inflammatory effects that may influence RA pathophysiology [10,23,24,25,26,27]. Vitamin D and calcium are essential for maintaining bone health, while vitamin D also plays a key role in modulating innate and adaptive immune responses through the regulation of inflammatory pathways [28,29,30].

Nutritional deficiencies, commonly observed in individuals with RA due to reduced dietary intake, altered nutrient metabolism, chronic inflammation, and medication-related effects, may aggravate systemic inflammation, increase oxidative stress, contribute to fatigue and functional impairment, and reduce health-related quality of life [10,29,30]. Comprehensive tools like the dietary diversity score (DDS) assess overall dietary quality and predict micronutrient adequacy [31,32], while the Dietary Inflammatory Index (DII) quantifies the inflammatory potential of a diet [12]. Existing research, however, primarily focuses on isolated dietary components or nutrients. Few studies, and none from India, have examined the association between DII and RA [33,34,35]. The combined effects of dietary adequacy, diversity, and inflammatory potential on RA-related inflammation and progression remain underexplored, particularly in the context of Indian diets.

This study aims to evaluate nutritional adequacy, dietary diversity, and dietary inflammatory index among RA patients compared to healthy controls. The study also examined the associations of these dietary measures with RA-related autoantibody concentrations. We hypothesized that higher DII and lower DDS and MPA would be associated with increased odds of RA, and that these dietary measures would be associated with higher RF and anti-CCP concentrations.

## 2. Methodology

### 2.1. Study Setting and Population

This unmatched case–control study was conducted in Hyderabad, India, and approved by the Institutional Ethics Committees of the National Institute of Nutrition (NIN IEC: 03/01/2014) and Nizam’s Institute of Medical Sciences (NIMS IEC: EC/NIMS/1665/2015; PBAC No. 1122/15). A total of 615 participants aged 18–55 years were enrolled, including 301 newly diagnosed RA cases and 314 healthy controls. Participants were recruited between 16 June 2017 and 31 December 2020, and written informed consent was obtained from all participants before enrolment. All participants were of Indian ethnicity and were recruited from Hyderabad and surrounding urban areas of Telangana State, India.

RA cases were diagnosed according to the 2010 ACR/EULAR classification criteria and confirmed by a Rheumatologist. Definite RA was defined by the presence of synovitis in at least one joint and a total ACR/EULAR score of ≥6/10. Controls were selected from schools, institutions, and private organizations in Hyderabad because recruitment of family/relative controls was not feasible. Frequency matching by age (±5 years) and sex was used at recruitment. Healthy controls were free from major chronic conditions, including type 2 diabetes, hypertension, PCOD, severe anemia, thyroid dysfunction, and autoimmune diseases, and were not pregnant or lactating. For both cases and controls, exclusion criteria included juvenile RA, seronegative spondyloarthritis, inflammatory bowel disease, celiac disease, malabsorption disorders, and current use of antibiotics, probiotics, or immunosuppressive treatment. Participants with BMI < 18.5 kg/m^2^ or >30 kg/m^2^ were also excluded.

### 2.2. Anthropometric Measurements and Dietary Assessment

Body weight was measured to the nearest 0.1 kg using a SECA scale, and standing height was measured to the nearest 0.5 cm using a stadiometer. BMI was calculated as weight (kg)/height (m^2^).

Dietary intake was assessed at enrolment through a single face-to-face interview conducted by trained nutritionists using a validated semi-quantitative food frequency questionnaire (FFQ) comprising 131 food items classified into 13 food groups according to the Indian Food Composition Tables (IFCT-2017) [36]. Each interview lasted approximately 40 min. Interviewers were trained in FFQ administration, portion-size estimation, and conversion of household measures into quantities of foods consumed. Twelve standardized cups ranging from 30 to 1400 mL were used as portion-size aids. The same dietary assessment procedure was followed for cases and controls. Interviewers were not blinded to participants’ case/control status.

The FFQ assessed habitual dietary intake over a 12-month period. Participants reported the usual frequency and quantity of consumption of each food item, categorized as daily, weekly, monthly, or less frequent/yearly intake, which was subsequently converted to estimated average daily intake. For RA cases, the 12-month reference period was anchored to the reported onset of symptoms/diagnosis, whereas for controls it referred to the 12 months preceding the interview. Reported portion sizes were converted to weight equivalents (g or mg). For mixed dishes, the quantity of each raw ingredient consumed was estimated as: Quantity of raw ingredient consumed = (Quantity of raw ingredient used ÷ Total quantity of cooked food) × Quantity of cooked food consumed.

### 2.3. Estimation of Nutrient Adequacy

Macronutrient adequacy was assessed using the acceptable macronutrient distribution range (AMDR) approach. For each participant, the percentage of total daily energy contributed by carbohydrate, fat, and protein was calculated and compared with the respective AMDRs.

Micronutrient adequacy was assessed using the probability of adequacy (PA) approach based on the estimated average requirement (EAR), which is recommended for population-level assessment of dietary adequacy.

PA was calculated for 10 micronutrients: iron, zinc, thiamine, riboflavin, niacin, vitamin B12, vitamin B6, vitamin C, vitamin A, and folate, for which the required EAR values and corresponding coefficient of variation (CV)-based requirement standard deviation (SD) inputs were available and applied in our analysis. Micronutrients without the required inputs for the PA approach were not included in the mean probability of adequacy (MPA) calculation.

For each micronutrient, the requirement SD was derived using the formula: SD = CV × EAR. The PA for each nutrient was then estimated using the EAR values provided by ICMR–NIN and the standard normal distribution function (NORM.S.DIST) in Microsoft Excel, representing the probability that an individual’s intake met the nutrient requirement. The Mean Probability of Adequacy (MPA) was calculated as the arithmetic mean of the probability of adequacy (PA) values for the 10 selected micronutrients. An MPA < 0.5 was used as an operational criterion to indicate overall micronutrient inadequacy in the study population [32,37,38,39].

### 2.4. Assessment of Dietary Diversity

The dietary diversity score (DDS) is a measure used to evaluate how diverse and nutritious an individual’s diet is. It shows the range of different food groups consumed within a specific time frame. Higher DDS values indicate better nutrient intake and overall health outcomes. Based on recent dietary guidelines 2024, the diet is divided into 10 food groups: cereals and millets, pulses, vegetables, nuts and oil seeds, green leafy vegetables, fruits, dairy, roots and tubers, flesh food and spices, and condiments [15]. A score of ‘1’ was assigned if a food group was consumed and ‘0’ otherwise. The overall dietary diversity score (DDS) was calculated by summing the scores across all food groups. Participants consuming fewer than five food groups (DDS < 5) were classified as having low dietary diversity, whereas those consuming five or more food groups (DDS ≥ 5) were classified as having high dietary diversity [31].

### 2.5. Assessment of Dietary Inflammatory Index (DII) Score

The FFQ-derived dietary data were utilized to compute the DII scores for each participant. A detailed explanation of the development and construct validation of the DII has been documented elsewhere. Following the approach described by Shivappa et al. [12], a counting algorithm was applied, based on an extensive literature review assessing the impact of diet on inflammatory biomarkers, including IL-4, TNF-α, IL-1β, IL-6, IL-10, and CRP. In this methodology, a total of 45 dietary components, encompassing both micronutrients and macronutrients, were assigned a score of (−1) for anti-inflammatory effects, (+1) for pro-inflammatory effects, or (0) if they had no impact on these inflammatory biomarkers.

In this study, dietary data from 36 dietary components collected through the FFQ were available for DII calculation. These dietary components included: Energy, Iron, Protein, Zinc, Carbohydrates, Thiamine, Fats, Riboflavin, MUFA, Vitamin B12, PUFA, Vitamin C, Trans fats, Vitamin A, Fiber, Vitamin D3, Folic acid, Vitamin B6, β-carotene, Total cholesterol, Vit E, Magnesium, Niacin, Selenium, Garlic, *n*-3 Fatty acids, Ginger, *n*-6 Fatty acids, Turmeric, Onion, Flavonones, Saturated Fat, Isoflavones, Pepper, Flavones, Flavan-3-ol (Supplementary Table S1). Each food constituent was assigned a DII score based on a global standard database. This score was calculated by subtracting the global mean from the reported intake and dividing by the standard deviation, resulting in a Z-score. To account for the right-skewed distribution commonly observed in dietary data, the Z-score was converted into a centered percentile score. Each dietary component’s centered percentile score was then multiplied by its respective dietary component-specific inflammatory effect score, as determined from literature reviews. The final DII score for each participant was obtained by summing all dietary component-specific DII scores, providing an overall measure of dietary inflammatory potential.

### 2.6. Antibody Measurement

Non-fasting venous blood samples (approximately 10 mL per participant) were collected by trained phlebotomists using aseptic techniques and sterile vacutainer tubes with appropriate anticoagulants (EDTA for plasma; serum separator tubes for serum). Serum and plasma were separated within 2 h of collection by centrifugation (1500× *g* for 10 min) and stored in aliquots at −80 °C until laboratory analysis (within 6 months). Serum Rheumatoid Factor (RF) and Plasma anti-cyclic citrullinated peptide (anti-CCP) levels were performed using ELISA technique as per manufacturer instructions (RF was detected by IgM RF ELISA, APSLABS, Pune, India), values were interpreted in IU/mL. Anti-CCP antibodies were detected by ELISA using commercially available kits (DRG, Marburg, Germany) and values were interpreted in U/mL.

### 2.7. Statistical Analysis

Continuous data were expressed as the median and interquartile range (IQR) and compared between cases and controls using the Mann–Whitney U test. Categorical data were presented as frequencies and percentages and analyzed using Pearson’s Chi-square test to assess differences between the two groups. To evaluate the associations of DDS, DII, and MPA with RA after adjustment for available covariates, we conducted separate unconditional logistic regression models including age, sex, and BMI. Unconditional logistic regression was chosen because complete frequency matching based on age and sex was not fully achieved. Age, sex, and BMI were included as covariates in the primary models. However, adjustment was limited to these available variables, and residual confounding by unmeasured factors could not be excluded. Multiple linear regression models adjusted for age, sex, and BMI were used to assess the associations of DDS, DII, and MPA with anti-CCP and RF concentrations. Additionally, we created the tertiles of DII and assessed its relationship with RA, anti-CCP and RF antibodies using appropriate regression models. Age, sex and BMI were considered potential confounders in the primary models. Because BMI may also be influenced by early RA or disease-related changes in appetite and body composition, sensitivity analyses were performed using models adjusted for age and sex without BMI.

Additionally, because several nutrient-specific PA distributions showed pronounced floor or ceiling effects, an exploratory sensitivity analysis was conducted using a restricted MPA that excluded thiamine, riboflavin, vitamin B12, vitamin B6, and folate. The restricted MPA was calculated from iron, zinc, niacin, vitamin C, and vitamin A, and its associations with RA status and autoantibody concentrations were evaluated using the same regression models as the primary analysis.

As an exploratory secondary analysis, population attributable fractions (PAFs) were estimated for higher DII, low DDS (DDS < 5.0 vs. DDS ≥ 5.0), and inadequate MPA (MPA < 0.5 vs. MPA ≥ 0.5). For PAF estimation related to DII, tertiles 2 and 3 were combined and compared to tertile 1. To calculate PAF for odds ratios (OR) > 1.0 and OR < 1.0, we used Levin’s formula: [PAF = P_exposed_ × (OR − 1) ÷ (1 + P_exposed_ × (1 − OR))] and [PAF = P_exposed_ × (1 − OR) ÷ (1 − P_exposed_ * (1 − OR))], respectively. The Wald method was used to estimate 95% confidence intervals for the PAFs. These estimates were calculated under a hypothetical causal framework and are presented for descriptive purposes only. Given the case–control design and the possibility of residual confounding, selection bias, reverse causation, and uncertain temporality, the assumptions required for causal interpretation of PAFs are not established in this study.

The associations of DII, DDS, and MPA with the RA status were considered the principal analyses. Analyses involving anti-CCP and RF concentrations, including categorical exposure comparisons, were treated as secondary/exploratory. No formal adjustment for multiple comparisons was applied; therefore, *p*-values from these secondary analyses are presented as nominal and should be interpreted cautiously.

All tests were two-sided, and *p*-values <0.05 was considered statistically significant. No formal adjustment for multiple comparisons was applied.

## 3. Results

### 3.1. Participant Characteristics and Nutrient Intake

The median age of the participants was 38.0 years (IQR: 32.0–45.0), with cases being significantly older than controls (41.0 vs. 35.0 years, *p* < 0.001). Females constituted the majority of the study population (88.6%), with comparable proportions in cases (87.0%) and controls (90.1%) (*p* = 0.486). The median BMI was significantly lower among cases compared to controls (23.14 vs. 25.62 kg/m^2^, *p* < 0.001). Cases reported consistently lower intake of macronutrients compared to controls, including energy (1908.22 vs. 2138.95 kcal), protein (42.75 vs. 50.93 g), carbohydrates (235.35 vs. 266.20 g), fats (71.64 vs. 84.54 g), and fiber (21.38 vs. 28.00 g), all *p* < 0.001. Similarly, the median intake of several micronutrients was significantly lower among cases, including iron (8.85 vs. 12.25 mg), zinc (5.91 vs. 6.53 mg), thiamine (0.49 vs. 0.68 mg), riboflavin (0.37 vs. 0.50 mg), niacin (7.48 vs. 8.84 mg), vitamin B6 (0.30 vs. 0.43 mg), vitamin C (72.52 vs. 98.22 mg), vitamin A (390.94 vs. 509.40 RE), beta-carotene (350.89 vs. 577.53 mcg), folate (180.89 vs. 238.68 mcg), selenium (2.35 vs. 4.52 mcg), vitamin E (0.65 vs. 1.00 mg), and magnesium (65.30 vs. 101.89 mg), all *p* < 0.001. However, no significant differences were observed for vitamin B12 and vitamin D intake (Table 1).

Additionally, cases had lower intake of total cholesterol (16.88 vs. 30.11 mg) and trans fats (0.89 vs. 1.60 g) compared to controls (*p* < 0.001). Intake of fatty acids, including MUFA (0.12 vs. 0.26 g), PUFA (0.35 vs. 0.50 g), *n*-3 fatty acids (0.10 vs. 0.13 g), and *n*-6 fatty acids (0.24 vs. 0.40 g), was also significantly lower among cases. Furthermore, consumption of bioactive compounds such as flavones, flavanones, flavan-3-ols, and isoflavones, as well as spices like onion, ginger, turmeric, and pepper, was significantly lower in cases compared to controls (Table 1).

### 3.2. Macronutrient and Micronutrient Adequacy

According to AMDR guidelines, macronutrient adequacy was suboptimal across both groups, with consistently lower adequacy observed among cases. A significantly lower proportion of cases met the carbohydrate AMDR compared to controls (41.5% vs. 50.3%, *p* = 0.029). Protein adequacy was also low in both groups, with only 19.9% of cases meeting the AMDR compared to 28.0% of controls (*p* = 0.019). Similarly, fat intake within the AMDR was slightly but significantly lower among cases (80.0%) compared to controls (86.3%) (*p* = 0.049). Energy adequacy was also lower in cases (49.5%) relative to controls (57.6%) (*p* = 0.043). Fiber intake was notably lower among cases, with median intake at 76.34% of EAR (IQR: 46.68, 115.84) compared to 100.37% (IQR: 62.17, 147.57) in controls (*p* < 0.001). Correspondingly, a significantly smaller proportion of cases met fiber adequacy (71.1%) compared to controls (88.5%) (*p* < 0.001) (Table 2).

The PA for several micronutrients was generally low, particularly among cases. Median PA for iron was substantially lower in cases compared to controls (0.13 vs. 0.99, *p* < 0.001). Similarly, zinc (0.00 vs. 0.01, *p* < 0.001) and niacin (0.01 vs. 0.04, *p* < 0.001) showed significantly lower adequacy among cases. For thiamine, riboflavin, vitamin B6, and vitamin B12, the median PA was near zero in both groups, indicating widespread inadequacy. In contrast, the probability of adequacy for vitamin C was high in both groups but remained significantly lower among cases (0.99 vs. 1.00, *p* < 0.001). Vitamin A adequacy was also significantly lower in cases compared to controls (0.05 vs. 0.28, *p* < 0.001), while folate adequacy was uniformly high across both groups. Overall, the median MPA was significantly lower among cases compared to controls (18.88% vs. 32.87%, *p* < 0.001). Correspondingly, only 21.3% of cases were nutritionally adequate compared to 34.7% of controls (*p* < 0.001), highlighting poorer overall nutrient adequacy among cases (Table 2). Although several micronutrients showed median PA values close to zero, meaningful between-group differences remained evident for MPA and selected micronutrients (Table 2).

### 3.3. DDS, DII and Antibody Distributions

The median DDS differed significantly between the cases and controls, with lower diversity observed among cases (5.0 [3.0, 6.0]) compared to controls (5.0 [4.0, 7.0]) (*p* < 0.001). A smaller proportion of cases achieved a high DDS (≥5), with 50.2% classified as having high DDS compared to 74.2% among controls (*p* < 0.001) (Table 3).

The DII scores ranged from −7.18 (maximally anti-inflammatory) to +4.93 (maximally pro-inflammatory). The median DII score was significantly higher among cases (2.18 [−0.43, 3.81]) compared to controls (0.37 [−2.19, 2.73]) (*p* < 0.001). Additionally, biomarkers of disease activity were markedly elevated among cases. Median anti-CCP levels were substantially higher in the cases (98.6 [15.1, 647.9] EU/mL) compared to controls (9.2 [3.72, 16.3] EU/mL). Similarly, RF levels were significantly elevated among cases (385.5 [139.0, 825.6] U/mL) relative to controls (12.6 [7.3, 17.3] U/mL) (*p* < 0.001 for both comparisons) (Table 3).

### 3.4. Adjusted Associations

After adjusting for age, sex, and BMI, each one-unit increase in the DII was associated with 17% higher odds of RA [AOR (95% CI): 1.17 (1.10, 1.24)]. The odds of RA increased across tertiles of DII (*p*-trend < 0.001), with participants in tertile 2 and tertile 3 having 1.86-fold [AOR (95% CI): 1.21, 2.85] and 2.55-fold [AOR (95% CI): 1.65, 3.93] higher odds of RA, respectively, compared to tertile 1. A one-unit increase in the DDS was associated with a 27% lower likelihood of RA [AOR (95% CI): 0.73 (0.66, 0.81)]. Participants with high DDS had significantly lower odds of RA compared to those with low DDS, corresponding to a 63% reduction in odds [AOR (95% CI): 0.37 (0.26, 0.54)]. Each 10% increase in the MPA of nutrients was associated with a 12% reduction in the odds of RA [AOR (95% CI): 0.88 (0.83, 0.94)]. Participants with adequate MPA had 46% lower odds of RA compared to those with inadequate MPA [AOR (95% CI): 0.54 (0.37, 0.80)] (Table 4).

In exploratory PAF analyses, the estimated fractions were 43.8% (95% CI: 24.6, 58.0) for higher DII, 39.5% (25.3, 49.9) for low DDS, and 37.7% (14.7, 54.6) for inadequate MPA (Supplementary Table S2). These estimates are descriptive and should not be interpreted as causal or as proportions of RA cases that could be prevented by modifying these dietary exposures (Supplementary Table S2).

Regarding antibody levels, DII (per unit increase) was not significantly associated with anti-CCP [β-coeff. (95% CI): 5.18 (−5.21, 15.58)] or RF levels [7.91 (−4.20, 20.01)]. However, compared to the lowest tertile, participants in DII tertile 2 had significantly higher anti-CCP levels [β-coeff. (95% CI): 76.01 (2.26, 149.76)], while no significant associations were observed for tertile 3 or for RF levels. Each one-unit increase in DDS was associated with significantly lower anti-CCP levels [β-coeff. (95% CI): −22.25 (−38.82, −5.69)], although its association with RF was not statistically significant. Similarly, a high DDS status was not significantly associated with anti-CCP or RF levels. MPA (per 10% increase) and MPA status were not significantly associated with anti-CCP or RF antibody levels, although the direction of association suggested lower antibody levels with higher nutrient adequacy (Table 4).

Sensitivity analyses excluding BMI from the adjustment set produced very similar estimates. For DII, DDS and MPA, the age- and sex-adjusted ORs were 1.18 (95% CI: 1.11, 1.26), 0.74 (0.67, 0.82), and 0.88 (0.83, 0.93), respectively, compared with BMI-adjusted ORs of 1.17 (1.10, 1.24), 0.73 (0.66, 0.81), and 0.88 (0.83, 0.94), respectively (Supplementary Table S3).

Similarly, the restricted MPA produced findings similar to those of the primary MPA. Each 10-percentage-point increase in restricted MPA was associated with lower odds of RA [AOR 0.90; 95% CI: 0.86, 0.95], compared with an AOR of 0.88 (0.83, 0.94) for the original MPA. Adequate restricted MPA was also associated with lower odds of RA [AOR 0.63; 95% CI: 0.44, 0.89], compared with 0.54 (0.37, 0.80) for the original MPA. Neither measure was significantly associated with anti-CCP or RF (Supplementary Table S4).

## 4. Discussion

In this case–control study, greater dietary inflammatory potential, lower dietary diversity, and lower micronutrient adequacy were associated with higher odds of RA after adjustment for age, sex, and BMI. Each one-unit increase in DII was associated with 17% higher odds of RA, whereas each one-unit increase in DDS and each 10-percentage-point increase in MPA were associated with 27% and 12% lower odds, respectively. Exploratory PAF estimates were 43.8% for higher DII, 39.5% for low DDS, and 37.7% for inadequate MPA. These estimates should be interpreted cautiously and only as model-based descriptive quantities under a hypothetical causal framework. Because the present case–control study cannot establish temporality or causality and may be affected by residual confounding and selection bias, the PAFs should not be interpreted as estimates of preventable RA burden.

Associations with RA-related autoantibodies were less consistent. Continuous DII was not associated with anti-CCP or RF, and although DII tertile 2 was associated with higher anti-CCP, this was not seen in tertile 3, indicating no clear dose–response pattern. Higher continuous DDS was associated with lower anti-CCP, but the categorical DDS analysis was not significant, and no association with RF was observed. MPA was not associated with either anti-CCP or RF. These autoantibody findings should therefore be considered secondary and interpreted cautiously.

Our findings are consistent with previous studies reporting associations between dietary patterns, inflammatory markers, and RA. Gao et al. [33] reported higher odds of RA in individuals consuming pro-inflammatory diets, with the highest DII tertile associated with elevated disease risk. Similarly, Nayebi [34] and Tandorost [40] observed that higher DII scores correlated with increased serum inflammatory markers (e.g., hs-CRP, TNF-α) and worse clinical outcomes (e.g., DAS-28 score). Evidence from Japan [41] and the UK Biobank [42] further supports the association between higher DII scores and increased RA activity and inflammation. In Japan, the TOMORROW study reported that RA patients had significantly higher energy-adjusted DII (E-DII) scores compared to controls, suggesting a more pro-inflammatory dietary pattern. While the E-DII score itself was not directly linked to significant changes in disease activity, a shift towards a more anti-inflammatory diet was associated with the maintenance of low disease activity over a six-year follow-up period. These findings support further investigation of dietary inflammatory potential in relation to RA progression [41].

Similarly, data from the UK Biobank revealed a significant association between diet, inflammation (measured by CRP levels), and RA, suggesting that higher DII scores are linked to increased RA activity and inflammation [42]. Conversely, anti-inflammatory dietary patterns characterized by high consumption of fruits, vegetables, whole grains, legumes, and omega-3 fatty acids have been shown to reduce systemic inflammation, improve disease activity, and alleviate clinical symptoms in patients with rheumatoid arthritis [10,11,13,14,43].

Moreover, studies on dietary patterns indicate that adherence to anti-inflammatory diets, such as Mediterranean or vegan diets, can reduce systemic inflammation and RA disease activity [44,45]. For example, randomized controlled trials have demonstrated that omega-3 supplementation reduces RA-related inflammation, while diets high in sugar, red meat, and saturated fats exacerbate disease progression [44,45,46]. Our study extends these findings by showing associations of dietary inadequacy and lower DDS with RA status, while associations with autoantibody concentrations were limited and inconsistent.

A plausible alternative explanation for the observed associations could be reverse causation, whereby early RA symptoms (e.g., pain, stiffness, fatigue, reduced appetite, and difficulty preparing or consuming certain foods) may have influenced food choices and overall intake before or around diagnosis, rather than diet alone contributing to disease risk. RA-related symptom burden may lead to lower energy intake, reduced dietary diversity, and poorer micronutrient adequacy, which could partly explain the more pro-inflammatory dietary profile observed in cases. In addition, health-seeking behavior after symptom onset may have prompted selective dietary changes or differential recall of “pre-disease” diet among cases compared with controls. Although we attempted to reduce this concern by recruiting newly diagnosed RA cases and assessing habitual intake using a validated interviewer-administered FFQ, these alternative explanations cannot be excluded in a case–control design and should be considered when interpreting the findings.

### 4.1. Potential Biological Mechanisms Linking Dietary Inflammatory Potential and RA

Several biological pathways may plausibly link dietary inflammatory potential with RA. Pro-inflammatory dietary patterns rich in saturated fats, refined carbohydrates, and processed foods promote systemic inflammation by enhancing the production of pro-inflammatory cytokines, including interleukin-6 (IL-6) and tumor necrosis factor-α (TNF-α), which play central roles in RA pathogenesis [10,20]. These cytokines are central mediators of RA pathogenesis, promoting persistent synovial inflammation, pannus formation, cartilage degradation, bone erosion, and the maintenance of autoimmune responses [47,48].

High DII scores, reflecting poor dietary quality, can also exacerbate oxidative stress and impair the body’s antioxidant defense systems, further promoting inflammatory cascades. Dietary patterns may influence RA pathogenesis through modulation of the gut microbiota. Pro-inflammatory dietary patterns are associated with gut dysbiosis, impaired intestinal barrier function, and heightened immune activation, whereas diets rich in dietary fiber, omega-3 fatty acids, and polyphenols promote microbial diversity, increase the production of anti-inflammatory metabolites such as short-chain fatty acids, and may contribute to reduced systemic inflammation and autoimmunity [10,11,17,18,43,49,50].

Micronutrient inadequacy may be relevant to RA-related inflammatory processes, although the present study cannot establish a causal contribution to disease progression. It may increase oxidative stress and antioxidant defense mechanisms. Deficiencies in vitamins B6, C, and E, along with trace minerals such as zinc and selenium, have been associated with increased oxidative stress and heightened inflammatory responses in RA [10,19,23,24,25]. Adequate intake of these micronutrients is critical for maintaining immune homeostasis and mitigating RA-associated inflammation.

### 4.2. Public Health Relevance

RA is a chronic autoimmune disease associated with substantial physical, social, and economic burden. In the present study, greater dietary inflammatory potential, lower dietary diversity, and lower micronutrient adequacy were associated with higher odds of RA. These findings suggest that overall dietary quality may be relevant to RA and provide a basis for further investigation of dietary patterns in relation to disease development and progression.

From a broader public health perspective, promoting diverse and nutritionally adequate diets rich in fruits, vegetables, whole grains, legumes, nuts, and other minimally processed foods is consistent with general recommendations for supporting overall health and adequate nutrient intake. The observed associations also suggest that dietary inflammatory potential, dietary diversity, and micronutrient adequacy may be useful areas for future nutritional research in populations at risk of RA.

However, the present case–control study cannot establish that modifying DII, DDS, or MPA would prevent RA, reduce disease severity, or improve clinical outcomes. The findings should therefore not be interpreted as evidence for specific dietary strategies for RA prevention or treatment. Prospective cohort studies are needed to establish temporality, while randomized dietary intervention trials are required to determine whether improving dietary quality or reducing dietary inflammatory potential has a measurable effect on RA development, disease activity, or related clinical outcomes.

### 4.3. Strengths and Limitations

Our study has several strengths. First, the relatively large sample size (615 participants) allowed us to examine the associations between dietary exposures and RA with reasonable precision while adjusting for the available covariates of age, sex, and BMI. Second, diet was assessed using a validated FFQ adapted with locally collected recipes, which improves the relevance of dietary intake estimates in this setting. The simultaneous evaluation of DII, DDS, and MPA has rarely been reported in South Asian populations.

In addition, RA cases were clinically diagnosed, and both anti-CCP and RF were measured, allowing assessment of associations with disease status as well as autoantibody levels. To our knowledge, this is among the first studies from India to jointly examine DII, DDS, and MPA in relation to RA and RA-related autoantibodies in a case–control design.

Despite its strengths, this study has several limitations. First, complete age/sex frequency matching was not achieved, and cases and controls differed in age and BMI. In addition, cases and controls were recruited from different settings; controls were recruited from schools, institutions, and private organizations because recruitment of healthy relatives was not feasible. This may have introduced selection bias and reduced comparability between groups. Although the regression models adjusted for age, sex, and BMI, residual differences and unmeasured confounding may remain.

Second, dietary intake was assessed retrospectively using an interviewer-administered FFQ. Although the FFQ was validated for the local population, recall error and exposure misclassification are possible. Interviewers were not blinded to case/control status, which may also have introduced interviewer-related measurement bias. The exact onset of RA may have been difficult to identify because symptoms can develop gradually and precede diagnosis. Early symptoms such as pain, stiffness, fatigue, reduced appetite, or difficulty preparing food may therefore have altered dietary intake before the reported onset of disease, creating the possibility of reverse causation.

Third, detailed information on medication exposure before enrolment, including corticosteroid dose, duration, and timing, was not systematically available. Because corticosteroids and other medications may affect appetite, body weight, and dietary behavior, residual confounding related to prior medication exposure cannot be excluded. Data were also unavailable for several potentially important confounders, including socioeconomic status, education, physical activity, smoking, alcohol intake, and genetic susceptibility. Therefore, adjustment was limited to age, sex, and BMI, and substantial residual or unmeasured confounding may remain. The adjusted associations should consequently not be interpreted as effects independent of these unmeasured factors.

Fourth, the DII was calculated using 36 of the original 45 dietary components because information on the remaining components was unavailable. Several excluded components have anti-inflammatory effect scores, and their absence may have affected the distribution of DII values and introduced exposure misclassification. However, the use of a reduced number of DII components is not unique to the present study; previous RA studies have calculated DII using 32 and 28 of the original 45 components, respectively [34,35]. Nevertheless, the present DII findings should be interpreted specifically in the context of the 36-component index used in this study.

Fifth, MPA was calculated using 10 micronutrients for which the required EAR and coefficient-of-variation inputs were available. Several nutrient-specific PA distributions showed pronounced floor or ceiling effects, which may have reduced their discriminatory contribution to the composite MPA. Although a restricted MPA sensitivity analysis excluding these nutrients produced broadly similar associations with RA, this does not constitute independent validation of the MPA measure. The MPA findings should therefore be interpreted cautiously, with greater emphasis on the overall pattern of association rather than individual nutrient-specific PA estimates.

Finally, the case–control design does not establish temporality or causality. The reported PAF estimates should therefore be considered theoretical estimates based on causal assumptions and not direct estimates of preventable RA burden. The study was also conducted in an urban population in southern India, with a predominance of women, which may limit generalizability to other populations and settings.

## 5. Conclusions

In this case–control study, individuals with RA had lower dietary diversity and micronutrient adequacy and greater dietary inflammatory potential than healthy controls. Higher DII and lower DDS and MPA were associated with higher odds of RA after adjustment for age, sex, and BMI. Associations with RA-related autoantibody concentrations were limited and inconsistent, involving mainly selected analyses of anti-CCP, while no significant associations with RF were observed. Given the case–control design and the potential for recall bias, reverse causation, selection bias, and residual confounding from unmeasured socioeconomic, lifestyle, medication-related, and genetic factors, these associations should not be interpreted causally. Prospective cohort studies and dietary intervention trials are needed to establish temporality and determine whether modification of dietary quality or inflammatory potential influences RA development or clinical outcomes.

## Figures and Tables

**Table 1:** Demographic, body mass index and nutrient consumption distribution across participants.

Variables		Overall (*n* = 615)	Case (*n* = 301)	Control (*n* = 314)	*p*-Value
Age in years, median (IQR)		38.0 (32.0, 45.0)	41.0 (37.0, 47.0)	35.0 (29.0, 42.0)	<0.001
Sex	Females	545 (88.6)	262 (87.0)	283 (90.1)	0.486
	Males	70 (11.4)	39 (13.0)	31 (9.9)	
Food Habits	Vegetarian	78 (12.7)	35 (11.6)	43 (13.7)	0.261
	Ovo-Vegetarian	23 (3.7)	8 (2.7)	15 (4.8)	
	Non-Vegetarian	514 (83.6)	258 (85.7)	256 (81.5)	
BMI (kg/m^2^)		24.56 (21.40, 27.18)	23.14 (20.40, 26.44)	25.62 (22.72, 27.58)	<0.001
Macronutrient Intake					
Energy in kcal, median (IQR)		2045.94 (1414.64, 2910.31)	1908.22 (1279.43, 2731.57)	2138.95 (1540.01, 3019.88)	<0.001
Protein in g, median (IQR)		47.30 (30.22, 71.79)	42.75 (27.27, 63.53)	50.93 (34.63, 76.42)	<0.001
Carbohydrate in g, median (IQR)		249.72 (178.37, 369.95)	235.35 (167.86, 337.45)	266.20 (197.43, 397.46)	<0.001
Fats in g, median (IQR)		79.11 (51.90, 114.45)	71.64 (46.61, 106.55)	84.54 (59.07, 124.89)	<0.001
Fiber in g, median (IQR)		24.50 (15.66, 36.48)	21.38 (13.07, 31.56)	28.00 (17.95, 40.86)	<0.001
Micronutrient Intake					
Iron in mg, median (IQR)		10.50 (6.59, 17.57)	8.85 (5.24 15.28)	12.25 (7.81, 18.86)	<0.001
Zinc in mg, median (IQR)		6.23 (4.35, 9.22)	5.91 (3.82, 8.35)	6.53 (4.71, 10.11)	<0.001
Thiamine in mg, median (IQR)		0.57 (0.33, 0.96)	0.49 (0.27, 0.80)	0.68 (0.42, 1.11)	<0.001
Riboflavin in mg, median (IQR)		0.44 (0.26, 0.72)	0.37 (0.21, 0.59)	0.50 (0.33, 0.81)	<0.001
Niacin in mg, median (IQR)		8.22 (5.32, 12.44)	7.48 (4.39, 10.94)	8.84 (6.17, 14.20)	<0.001
Vitamin B12 in mcg, median (IQR)		0.00 (0.00, 0.05)	0.00 (0.00, 0.05)	0.00 (0.00, 0.05)	0.259
Vitamin B6 in mg, median (IQR)		0.36 (0.19, 0.70)	0.30 (0.16, 0.55)	0.43 (0.23, 0.80)	<0.001
Vitamin C in mg, median (IQR)		87.39 (52.74, 157.57)	72.52 (41.12, 137.85)	98.22 (60.92, 166.39)	<0.001
Vitamin D in mg, median (IQR)		10.00 (4.93, 19.23)	9.87 (4.75, 17.60)	10.20 (5.13, 19.45)	0.499
Vitamin A in RE, median (IQR)		443.31 (266.30, 814.70)	390.94 (211.93, 706.45)	509.40 (312.92, 887.21)	<0.001
Beta carotene in mcg, median (IQR)		471.93 (212.37, 1075.38)	350.89 (166.57, 1063.00)	577.53 (270.45, 1154.24)	<0.001
Folate in mcg, median (IQR)		202.88 (126.31, 341.38)	180.89 (104.44, 290.64)	238.68 (146.14, 373.79)	<0.001
Total cholesterol in mg, median (IQR)		22.90 (11.2, 43.5)	16.88 (8.5, 34.06)	30.11 (15.28, 55.09)	<0.001
Selenium in mcg, median (IQR)		3.00 (1.35, 7.94)	2.35 (0.99, 5.27)	4.52 (1.96, 10.63)	<0.001
Vitamin E in mg, median (IQR)		0.79 (0.33, 1.81)	0.65 (0.26, 1.21)	1.00 (0.43, 2.48)	<0.001
Magnesium in mg, median (IQR)		82.49 (39.78, 159.60)	65.30 (30.99, 121.75)	101.89 (53.10, 202.48)	<0.001
Trans fats in g, median (IQR)		1.20 (0.49, 2.72)	0.89 (0.35, 2.24)	1.60 (0.79, 3.12)	<0.001
Isoflavones in mg, median (IQR)		0.00 (0.00, 0.002)	0.00 (0.00, 0.00)	0.00 (0.00, 0.008)	<0.001
Flavones in mg, median (IQR)		1.28 (0.66, 2.46)	0.98 (0.47, 1.95)	1.44 (0.87, 2.82)	<0.001
Flavanones in mg, median (IQR)		0.001 (0.000, 0.004)	0.001 (0.000, 0.002)	0.001 (0.000, 0.007)	<0.001
Flavan3ols in mg, median (IQR)		1.85 (0.78, 4.07)	1.37 (0.58, 3.40)	2.22 (1.08, 5.42)	<0.001
Garlic in g, median (IQR)		0.05 (0.02, 0.11)	0.05 (0.02, 0.12)	0.05 (0.02, 0.10)	0.486
Onion in g, median (IQR)		2.59 (1.03, 5.33)	2.01 (0.80, 4.55)	3.17 (1.42, 6.04)	<0.001
Ginger in g, median (IQR)		0.60 (0.33, 1.25)	0.49 (0.26, 1.07)	0.70 (0.42, 1.41)	<0.001
Turmeric in g, median (IQR)		0.06 (0.03, 0.13)	0.04 (0.02, 0.12)	0.07 (0.04, 0.14)	<0.001
Pepper in g, median (IQR)		0.07 (0.03, 0.13)	0.06 (0.03, 0.13)	0.08 (0.04, 0.13)	0.001
SFA in g, median (IQR)		0.74 (0.24, 2.54)	0.54 (0.21, 2.17)	0.90 (0.37, 2.60)	0.001
MUFA in g, median (IQR)		0.18 (0.07, 0.77)	0.12 (0.05, 0.67)	0.26 (0.09, 0.77)	<0.001
PUFA in g, median (IQR)		0.43 (0.20, 0.82)	0.35 (0.17, 0.64)	0.50 (0.22, 1.03)	<0.001
*n*-3 fatty acids in g, median (IQR)		0.12 (0.06, 0.23)	0.10 (0.05, 0.21)	0.13 (0.07, 0.25)	<0.001
*n*-6 fatty acids in g, median (IQR)		0.31 (0.15, 0.60)	0.24 (0.12, 0.45)	0.40 (0.17, 0.84)	<0.001

IQR, interquartile range; BMI, body mass index; mg, milligram; mcg, microgram; MUFA, monounsaturated fatty acids; PUFA, polyunsaturated fatty acids. Continuous variables are presented as median (interquartile range) and were compared between cases and controls using the Mann–Whitney U test. Categorical variables are presented as *n* (%) and were compared using Pearson’s chi-square test. All tests were two-sided.

**Table 2:** Acceptable macronutrient distribution range and probability of adequacy of micronutrients.

	Overall (*n* = 615)	Case (*n* = 301)	Control (*n* = 314)	*p*-Value
Acceptable Macronutrient Distribution Range				
Protein AMDR (Adequate), *n* (%)	148 (24.1)	60 (19.9)	88 (28.0)	0.019
Fat AMDR (Adequate), *n* (%)	513 (83.4)	242 (80.0)	271 (86.3)	0.049
Carbohydrate AMDR (Adequate), *n* (%)	283 (46.0)	125 (41.5)	158 (50.3)	0.029
Energy status (Adequate), *n* (%)	330 (53.7)	149 (49.5)	181 (57.6)	0.043
Fiber consumption (in % EAR), median (IQR)	85.37 (56.14, 128.55)	76.34 (46.68, 115.84)	100.37 (62.17, 147.57)	<0.001
Fiber consumption status (Adequate), *n* (%)	492 (80.0)	214 (71.1)	278 (88.5)	<0.001
Probability of Adequacy of Micronutrients				
Iron, median (IQR)	0.69 (0.00, 1.00)	0.13 (0.00, 1.00)	0.99 (0.01, 1.00)	<0.001
Zinc, median (IQR)	0.01 (0.00, 0.76)	0.00 (0.00, 0.38)	0.01 (0.00, 0.96)	<0.001
Thiamine, median (IQR)	0.00 (0.00, 0.33)	0.00 (0.00, 0.04)	0.00 (0.00, 0.88)	<0.001
Riboflavin, median (IQR)	0.00 (0.00, 0.00)	0.00 (0.00, 0.00)	0.00 (0.00, 0.01)	<0.001
Niacin, median (IQR)	0.02 (0.00, 0.60)	0.01 (0.00, 0.28)	0.04 (0.00, 0.89)	<0.001
Vitamin B12, median (IQR)	0.00 (0.00, 0.00)	0.00 (0.00, 0.00)	0.00 (0.00, 0.00)	0.551
Vitamin B6, median (IQR)	0.00 (0.00, 0.00)	0.00 (0.00, 0.00)	0.00 (0.00, 0.00)	<0.001
Vitamin C, median (IQR)	1.00 (0.11, 1.00)	0.99 (0.00, 1.00)	1.00 (0.56, 1.00)	<0.001
Vitamin A, median (IQR)	0.12 (0.01, 0.98)	0.05 (0.00, 0.86)	0.28 (0.01, 1.00)	<0.001
Folate, median (IQR)	1.00 (1.00, 1.00)	1.00 (1.00, 1.00)	1.00 (1.00, 1.00)	<0.001
MPA in %, median (IQR)	24.24 (4.63, 55.89)	18.88 (0.72, 43.18)	32.87 (12.51, 63.01)	<0.001
MPA status (Adequate), *n* (%)	173 (28.1)	64 (21.3)	109 (34.7)	<0.001

AMDR, acceptable macronutrient distribution range; IQR, interquartile range; MPA, mean probability of adequacy; *n*, frequency; %, percentage; EAR, estimated average requirement. Continuous variables are presented as median (interquartile range) and were compared between cases and controls using the Mann–Whitney U test. Categorical variables are presented as *n* (%) and were compared using Pearson’s chi-square test. All tests were two-sided.

**Table 3:** Distribution of mean probability of adequacy of micronutrients, dietary score and dietary inflammatory potential across participants.

	Overall (*n* = 615)	Case (*n* = 301)	Control (*n* = 314)	*p*-Value
DDS, median (IQR)	5.0 (4.0, 6.0)	5.0 (3.0, 6.0)	5.0 (4.0, 7.0)	<0.001
DDS status (High), *n* (%)	384 (62.4)	151 (50.2)	233 (74.2)	<0.001
DII, median (IQR)	1.52 (−1.57, 3.28)	2.18 (−0.43, 3.81)	0.37 (−2.19, 2.73)	<0.001
Anti-CCP in EU/mL, median (IQR)	15.8 (7.0, 100)	98.6 (15.1, 647.9)	9.2 (3.72, 16.3)	<0.001
RF in U/mL, median (IQR)	31.0 (12.36, 377.0)	385.5 (139.0, 825.6)	12.6 (7.3, 17.3)	<0.001

DDS, dietary diversity score; DDS ≥ 5 is considered High; IQR, interquartile range; *n*, frequency; %, percentage; RF, Rheumatoid Factor; Anti-CCP, anti-cyclic citrullinated peptide. Continuous variables are presented as median (interquartile range) and were compared between cases and controls using the Mann–Whitney U test. Categorical variables are presented as *n* (%) and were compared using Pearson’s chi-square test. All tests were two-sided.

**Table 4:** Adjusted associations of dietary inflammatory potential, dietary diversity and micronutrient adequacy with rheumatoid arthritis status and RA-related autoantibody concentrations.

Variables	Comparison/Scale	Rheumatoid Arthritis		Anti-CCP (EU/mL)		RF Antibodies (U/mL)	
		AOR (95% CI)	*p*-Value	β-Coeff (95% CI)	*p*-Value	β-Coeff (95% CI)	*p*-Value
DII, continuous	Per one-unit increase	1.17 (1.10, 1.24)	<0.001	5.18 (−5.21, 15.58)	0.328	7.91 (−4.20, 20.01)	0.200
DII Tertiles			<0.001 *		0.329 *		0.479 *
Tertile 1	Reference (−7.18 to −0.55)	1.00	–	0.00	–	0.00	–
Tertile 2	T2 vs. T1 (−0.52 to 2.677)	1.86 (1.21, 2.85)	0.005	76.01 (2.26, 149.76)	0.043	52.71 (−33.44, 138.86)	0.230
Tertile 3	T3 vs. T1 (2.679 to 4.93)	2.55 (1.65, 3.93)	<0.001	25.58 (−48.25, 99.41)	0.497	23.99 (−62.25, 110.24)	0.585
DDS, continuous	Per one-unit increase	0.73 (0.66, 0.81)	<0.001	−22.25 (−38.82, −5.69)	0.009	−11.25 (−30.65, 8.15)	0.255
High DDS status	≥5 vs. <5 (reference)	0.37 (0.26, 0.54)	<0.001	−36.99 (−99.39, 25.41)	0.245	−51.49 (−124.18, 21.20)	0.165
MPA, continuous	Per 10-percentage-point increase	0.88 (0.83, 0.94)	<0.001	−2.53 (−12.87, 7.80)	0.630	−6.15 (−18.18, 5.88)	0.316
Adequate MPA	≥50% vs. <50% (reference)	0.54 (0.37, 0.80)	<0.001	−20.48 (−87.91, 46.96)	0.551	−44.65 (−123.19, 33.88)	0.265

AOR, adjusted odds ratio; CI, confidence interval; DII, dietary inflammatory index; DDS, dietary diversity score; *n*, frequency; %, percentage; RF, Rheumatoid Factor; Anti-CCP, anti-cyclic citrullinated peptide; MPA, mean probability of adequacy; DDS ≥ 5 is considered High; MPA ≥ 0.5 considered nutrient adequacy. Regression models were adjusted for age, sex and body mass index; * *p*-trend across the tertiles of DII for all outcome variables. AORs for rheumatoid arthritis were estimated using separate unconditional logistic regression models. β coefficients for anti-CCP and RF concentrations were estimated using separate multiple linear regression models. Each model included the indicated dietary exposure and was adjusted for age, sex and BMI. For categorical analyses, the reference categories are indicated in the table. For DII tertiles, Tertile 1 was the reference category, and the *p*-trend was calculated across increasing DII tertiles. *p*-values for the autoantibody analyses are unadjusted for multiplicity and should be interpreted as exploratory.

## Data Availability

The original contributions presented in this study are included in the article/Supplementary Material. Further inquiries can be directed to the corresponding author.

## Supplementary Materials

The following supporting information can be downloaded at: https://www.mdpi.com/article/10.3390/nu18193170/s1, Table S1. Included and Excluded Dietary Components Used to Calculate the Dietary Inflammatory Index. Table S2. Population Attributable Fractions (PAFs), Exposure Prevalence, and Adjusted Odds Ratios for Incident Rheumatoid Arthritis. Table S3. Age- and sex-adjusted associations of dietary inflammatory potential, dietary diversity and micronutrient adequacy with rheumatoid arthritis status and RA-related autoantibody concentrations. Table S4. Adjusted associations of micronutrient adequacy and restricted micronutrient adequacy with rheumatoid arthritis status and RA-related autoantibody concentrations.
