# Supplementary material for: Associations of Dietary Inflammatory Potential, Dietary Diversity, and Micronutrient Adequacy with Rheumatoid Arthritis: A Case–Control Study from India
Source: Nutrients. 2026 Sep 26;18(19):3170. doi: 10.3390/nu18193170 (PMC13667051; doi:10.3390/nu18193170)
Supplement: Supplementary file 1 [file nutrients-18-03170-s001.zip › nutrients-4507138-supplementary.pdf]

Supplementary Table S1. Included and Excluded Dietary Components Used to Calculate the Dietary Inflammatory Index

Included Components	
Energy	Iron
Protein	**Zinc**
Carbohydrates	**Thiamine**
Fats	**Riboflavin**
**MUFA**	Vitamin B12
**PUFA**	**Vitamin C**
Trans fats	**Vitamin A**
**Fiber**	**Vitamin D3**
**Folic acid**	**Vitamin B6**
**β-carotene**	Total cholesterol
Vit E	**Magnesium**
Niacin	**Selenium**
**Garlic**	**n-3 Fatty acids**
**Ginger**	**n-6 Fatty acids**
**Turmeric**	**Onion**
**Flavanones**	Saturated Fat
**Isoflavones**	**Pepper**
**Flavones**	**Flavan-3-ol**
Excluded Components	
**Alcohol**	**Saffron**
**Caffeine**	**Green/black tea**
**Eugenol**	**Anthocyanidines**
**Flavonols**	**Rosemary**
**Thyme/oregano**	

Dietary components highlighted in bold are assumed to have an anti-inflammatory effect score

Supplementary Table S2. Population Attributable Fractions (PAFs), Exposure Prevalence, and Adjusted Odds Ratios for Incident Rheumatoid Arthritis

Exposure (risk category vs reference)	Reference category	Prevalence of exposure (Pe)	Adjusted OR (95% CI)	PAF (%)	95% CI
DII (T2+T3 vs T1)	T1 (lowest tertile)	66.70%	2.17 (1.49–3.16)	43.8	24.6–58.0
DDS (Low <5.0 vs High ≥5.0)	High DDS (≥5.0)	37.60%	2.70 (1.90–3.90)	39.5	25.3–49.9
MPA (Inadequate <50% vs Adequate ≥50%)	Adequate intake (≥50%)	71.90%	1.84 (1.24–2.72)	37.7	14.7–54.6

PAF estimates are exploratory and model-based. They are presented under a hypothetical causal framework and should not be interpreted as direct estimates of causal attribution or preventable RA burden;

OR = Odds Ratio adjusted for covariates (specify your model variables if needed); Pe = prevalence of exposure in the study population; PAF = Population Attributable Fraction calculated assuming a causal relationship; DII = Dietary Inflammatory Index; DDS = Dietary Diversity Score; MPA = Mean Probability of Adequacy; T= Tertile;

Unconditional Logistic Regression models were adjusted for age, sex and body mass index;

Supplementary Table S3. Age- and sex-adjusted associations of dietary inflammatory potential, dietary diversity and micronutrient adequacy with rheumatoid arthritis status and RA-related autoantibody concentrations

Variables		Rheumatoid Arthritis		Anti-CCP (EU/mL)		RF antibodies (U/mL)	
	Comparison/ scale	AOR (95% CI)	p-value	β-coeff (95% CI)	p-value	β-coeff (95% CI)	p-value
DII, continuous	Per 1-unit increase	1.18 (1.11, 1.26)	<0.001	7.42 (-3.05, 17.90)	0.164	11.26 (-1.04, 23.57)	0.073
DII Tertiles			<0.001 *		0.187*		0.252 *
Tertile 1	Reference (-7.18 to -0.55)	1.00	-	0.00	-	0.00	-
Tertile 2	T2 vs. T1 (-0.52 to 2.677)	2.02 (1.33, 3.07)	0.005	88.79 (14.34, 163.24)	0.020	72.21 (-15.57, 160.00)	0.107
Tertile 3	T3 vs. T1 (2.679 to 4.93)	2.69 (1.76, 4.10)	<0.001	37.65 (-36.91, 112.20)	0.322	42.41 (-45.51, 130.32)	0.344
DDS, continuous	Per 1-unit increase	0.74 (0.67, 0.82)	<0.001	-23.80 (-40.58, -7.03)	0.005	-13.61 (-33.44, 6.22)	0.178
High DDS status	≥5 vs <5 (reference)	0.36 (0.25, 0.52)	<0.001	-48.31 (-111.31, 14.68)	0.133	-68.49 (-142.53, 5.55)	0.070
MPA, continuous	Per 10-percentage-point increase	0.88 (0.83, 0.93)	<0.001	-4.61 (-15.03, 5.81)	0.386	-9.23 (-21.48, 3.02)	0.139
Adequate MPA	≥50% vs <50% (reference)	0.51 (0.35, 0.75)	0.001	-31.16 (-99.33, 37.01)	0.370	-60.59 (-140.69, 19.51)	0.138

AOR, adjusted odds ratio; CI, confidence interval; DII, dietary inflammatory index; DDS, dietary diversity score; IQR, interquartile range; n, frequency; %, percentage; RF, Rheumatoid Factor; Anti-CCP, anti-cyclic citrullinated peptide; MPA, mean probability of adequacy; ref, reference category; DDS score ≥5 is considered as *High*; MPA ≥0.5 considered as nutrient adequacy;

Regression models were adjusted for age and sex; *p-trend across the tertiles of DII for all outcome variables.

AORs for rheumatoid arthritis were estimated using separate unconditional logistic regression models. β coefficients for anti-CCP and RF concentrations were estimated using separate multiple linear regression models. Each model included the indicated dietary exposure and was adjusted for age and sex. For categorical analyses, the reference categories are indicated in the table. For DII tertiles, Tertile 1 was the reference category, and the p-trend was calculated across increasing DII tertiles.

Supplementary Table S4. Adjusted associations of micronutrient adequacy and restricted micronutrient adequacy with rheumatoid arthritis status and RA-related autoantibody concentrations

Variables		Rheumatoid Arthritis		Anti-CCP (EU/mL)		RF antibodies (U/mL)	
	**Comparison/ scale**	**AOR (95% CI)**	**p-value**	**β-coeff (95% CI)**	**p-value**	**β-coeff (95% CI)**	**p-value**
MPA, continuous	Per 10-percentage-point increase	0.88 (0.83, 0.94)	<0.001	-2.53 (-12.87, 7.80)	0.630	-6.15 (-18.18, 5.88)	0.316
Adequate MPA	≥50% vs <50% (reference)	0.54 (0.37, 0.80)	<0.001	-20.48 (-87.91, 46.96)	0.551	-44.65 (-123.19, 33.88)	0.265
MPA-Res, continuous	Per 10-percentage-point increase	0.90 (0.86, 0.95)	<0.001	-2.03 (-10.54, 6.48)	0.640	-4.30 (-14.22, 5.61)	0.394
Adequate MPA-Res	≥50% vs <50% (reference)	0.63 (0.44, 0.89)	<0.001	-22.11 (-83.14, 38.91)	0.477	20.24 (-50.89, 91.37)	0.576

Restricted micronutrient adequacy (MPA-Res) was based on iron, zinc, niacin, vitamin C, and vitamin A
